# Use of 1,470 nm laser for treatment of superficial venous insufficiency

**DOI:** 10.1590/1677-5449.200244

**Published:** 2021-06-25

**Authors:** Manuella Bernardo Ferreira, Gilberto do Nascimento Galego, Nazaré Otília Nazário, Rafael Narciso Franklin, Pierre Galvagni Silveira, Cristiano Torres Bortoluzzi, Daniel Ishikawa, Fernando Wolf

**Affiliations:** 1 Universidade do Sul de Santa Catarina – UNISUL, Palhoça, SC, Brasil.; 2 Universidade Federal de Santa Catarina – UFSC, Florianópolis, SC, Brasil.; 3 Clínica Coris Medicina Vascular do Baía Sul Medical Center, Florianópolis, SC, Brasil.

**Keywords:** venous insufficiency, varicose veins, laser angioplasty, intravenous ablation

## Abstract

**Background:**

There are several ways to treat varicose veins of the lower limbs, among which use of 1470nm diode lasers stands out. This technique can be used to treat patients in outpatient settings, with early return to work, good esthetic results, and low rates of complications. However, variables such as the laser wavelength, the power administered in each area, the type of fiber, and the linear intravenous energy density (LEED) are still extensively discussed.

**Objectives:**

To analyze the results of superficial venous insufficiency treatment with a 1470nm diode laser.

**Methods:**

Retrospective study conducted at a private clinic in a private hospital in Florianopolis, based on a database collected prospectively. The sample comprised 287 patients who underwent surgery to treat superficial venous insufficiency with 1470nm diode laser, from January 2016 to December 2018, totaling 358 great saphenous veins (GSVs) and 84 small saphenous veins (SSVs) treated.

**Results:**

The total occlusion rates after 12 months of surgery were 94.4% in the GSVs, with an average LEED of 45.90 J/cm, and 96.4% in the SSVs, with an average LEED of 44.07 J/cm.

**Conclusions:**

During the follow-up period, the 1470nm diode laser proved to be a safe treatment, with great efficacy and low rates of complications (pain, edema, bruising, deep vein thrombosis, and endothermal heat-induced thrombosis - EHIT).

## INTRODUCTION

Chronic venous insufficiency (CVI) is defined as the set of clinical manifestations caused by hemodynamic disorders such as reflux and/or obstruction of the peripheral venous system (superficial and/or deep), generally involving the lower limbs.^1^ It is estimated that half of the global population has reticular veins and telangiectasies of the lower limbs, while 25% have larger and more visible varicose veins.^2^ In Brazil, the prevalence of varicose veins varies from 41.2 to 62.7% among women and 13.9 to 37.9% among men.^3^ According to the Ministry of Health, the socioeconomic repercussions of CVI put it in 14th position among the 50 diseases that cause most absence from work.^4^^,^^5^

This high prevalence is associated with aging, obesity, family history, female sex (5:1), Caucasian women, use of oral contraception, hormone replacement therapy, and working standing up.^2^^,^^3^ Clinical assessment to assess anatomic distribution and to quantify the hemodynamic effects is needed to define the severity of the disease, because of its impact in terms of reduced quality of life and the chronic and subjective nature of complaints and symptoms such as pain, feelings of heaviness, swelling, cramps, and others.^1^^,^^6^^,^^7^

Varicose veins are the most common manifestation of CVI. They start at points of reflux, such as at the saphenofemoral junction (SFJ), the saphenopopliteal junction (SPJ), or the perforating vein systems due to valve insufficiency and/or dilation of the vein walls. These events cause stasis, drainage deficiency, and venous hypertension, resulting in edema, impaired supply to tissues, and skin changes, which consequently predispose to inflammatory processes and infectious, increasing the risk of venous thromboses and lesions such as lipodermatosclerosis and venous ulcers.^2^^,^^7^^-^9

The standard intervention to avoid disease progression had been ligature and stripping of the great saphenous vein (GSV) and/or small saphenous vein (SSV) combined with exeresis of varicose veins and ligature of incompetent perforating veins.^10^ As treatments have developed, new approaches offering equivalent results and employing less invasive techniques have emerged, including endovenous laser treatment (EVLT), radio frequency, foam sclerotherapy, combined or not with phlebectomy, and liquid sclerotherapy.^10^^-^12

Of these, EVLT can be used to treat patients in ambulatory settings, enabling early return to work, better esthetic results, and low rates of complications. This technique employs endovenous laser to provoke venous ablation, treating reflux in the territory.^10^^,^^13^^-^16 However, there is endless debate on the effects of different types of fibers, wavelengths, and laser power and also on the linear intravenous energy density (LEED) that should be administered to achieve the best therapeutic results and minimize complications such as pain, ecchymosis, thrombophlebitis, endothermal heat-induced thrombosis (EHIT), and deep venous thrombosis (DVT). Against this background, the present study describes the results of treatment of superficial venous insufficiency with 1,470 nanometer (nm) diode laser.

## METHODOLOGY

A descriptive cross-sectional study of data from a database maintained by a private clinic specialized in endovascular treatments, located within a private hospital in Florianopolis, Santa Catarina, Brazil. The sample comprised 287 patients who underwent surgery for lower limb varicose veins with 1,470 nm diode laser and radial fiber, from January 2016 to December 2018. The sample of 280 patients was considered sufficient to measure an expected prevalence of 18% of greater severity, according to the Clinical, Etiological, Anatomic, and Pathological (CEAP) classification, with acceptable error of +/- 5%, a 95% confidence interval, and 80% power. Patients were included who were over the age of 18 years, of both sexes, with diagnosis and indications for surgical treatment for unilateral or bilateral lower limb varicose veins in CEAP classes C2 to C6, for whom a minimum of two control Doppler ultrasonography examinations had been performed during the postoperative period, and who had undergone ablation of the GSV and/or SSV with the 1,470 nm laser. Patients with a prior diagnosis of DVT were excluded.

The protocol employed comprises three stages. The first, preoperative stage is a clinical assessment with Doppler ultrasonography, enabling CEAP classification and mapping of venous reflux and the diameters of the vessels involved. In the second, transoperative stage, information collected in the preoperative stage is confirmed and, with the aid of Doppler ultrasonography, the venous puncture site is chosen. The fiber path and the results of ablation are monitored. The vascular surgeon defines the 1,470 nm laser power to be used on the basis of the diameter of the incompetent vessel and its proximity to the skin. The fiber is tractioned at constant velocity, releasing energy and causing venous ablation, while manual compression or compression with the Doppler ultrasonography transducer is applied to bring the vein walls into proximity with the fiber. In some cases, the tumescence technique is also employed. This consists of infiltration of chilled 0.9% saline with the aid of Doppler ultrasonography along the entire length of the saphenous vein until it collapses, with a blanched appearance or the pearl sign. Collaterals are resected and/or treated with sclerotherapy during the same surgical session. At the end of ablation, the total energy dissipated is noted on the protocol together with the length of vessel treated, enabling LEED to be calculated (Joules/cm). The decision of whether to Dissect and ligate the saphenous arch is taken by the surgeon, depending on the diameter of the saphenous vein at the level of the SFJ and SPJ and on data viewed with Doppler ultrasonography. Before ending the procedure, Doppler ultrasonography is used to confirm patency of the femoral and popliteal veins and confirm ablation of the saphenous vein(s). All patients are treated in an operating room, under spinal anesthesia.

During the third stage, follow-up, patients are told to walk as soon as possible and instructed to wear thigh-high elastic stockings and/or fully bandage the lower limbs. Between 7 and 10 days after the procedure, patients are examined to check for ecchymosis, pain, paresthesia, and edema and instructed to change to medium compression knee-high elastic stockings. Follow-up examinations with Doppler ultrasonography are conducted at 30 days, 6 months, and 12 months to assess the hemodynamic status of saphenous veins and check for complications (DVT, EHIT, etc.). If DVT is suspected or a clinical criterion is present (disproportional pain and edema, sudden onset edema, poor recovery, and others), the Doppler ultrasonography examination is conducted early. Treatment is defined as successful if there is total occlusion of the segment treated. During follow-up, evidence of flow through a venous segment previously defined as occluded in a previous Doppler ultrasonography examination is considered treatment failure and defined as recanalization.

Data were compiled using Excel^®^ 12.63 (Microsoft Corporation, Washington, United States) and exported to the Statistical Package for the Social Sciences 18.0 (IBM, New York, United States) for statistical analysis. For descriptive analysis, qualitative variables were expressed as simple and relative frequencies and quantitative variables were expressed using measures of central tendency and their respective measures of dispersion. The project was submitted to and approved by the Research Ethics Committee at the Universidade do Sul de Santa Catarina, under ruling number 3.494.758.

## RESULTS

From January 2016 to December 2018, 287 patients underwent varicose vein treatment with a 1,470 nm diode laser and radial fiber. The mean of age of the study population was 52.36 years and 219 (76.3%) patients were female. As indicated, the need for venous treatment was decided individually for each patient. The most prevalent interventions were ablation of just one GSV, in 136 (47.4%) cases, and ablation of two GSVs, in 78 (27.2%). Table 1 lists all of the demographic characteristics.

**Table 1:** Demographic data on patients (n = 287).

Mean age (years)	52.36 (19 to 77)
Sex	
Female	219 (76.3%)
Male	68 (23.7%)
ASA	
1	101 (38.4%)
2	161 (61.2%)
3	1 (0.4%)
Type of surgery	
1 x GSV	136 (47.4%)
2 x GSV	78 (27.2%)
1 x SSV	17 (5.9%)
2 x SSV	7 (2.4%)
1 x GSV & 1 x SSV	29 (10.1%)
2 x GSV & 1 x SSV	16 (5.6%)
1 x GSV & 2 x SSV	2 (0.7%)
2 x GSV & 2 x SSV	2 (0.7%)

ASA = American Society of Anesthesiologists; GSV = Great Saphenous Vein; SSV = Small Saphenous Vein.

With regard to patients’ CEAP class, it was observed that the majority of cases varied from C2 to C4. The most frequent class was C3, in which edema is present. This information is detailed in Table 2. Data on 30 patients are missing because the CEAP classification was not recorded on their medical charts.

**Table 2:** Preoperative clinical classification (CEAP).

	C1	C2	C3	C4	C5	C6
Overall (n = 442)	0	77	274	52	4	5
GSV (n = 358)	0	61	224	41	3	4
SSV (n = 84)	0	16	50	11	1	1

GSV = Great Saphenous Vein; SSV = Small Saphenous Vein.

Tables 3 and 4 list the data observed during preoperative Doppler ultrasonography, listed by region. The tables also list the mean power and LEED per area needed to achieve ablation during surgery. At the level of the thigh, the GSV had a mean diameter of 5.90 mm and mean power of 8.12 W was administered, calculating mean LEED at 52.85 J/cm. When total energy and length of ablation were calculated for the whole GSV, mean LEED was 45.90 J/cm. For the SSV, mean diameter at the proximal leg was 5.02 mm, mean power administered was 7.18 W, and mean LEED was 46.86 J/cm. Total mean LEED for the SSV was 44.07 J/cm.

**Table 3:** Preoperative and transoperative data on the great saphenous vein.

	Great saphenous vein								
	Preoperative			Transoperative					
	Mean Ø (mm)	Variation (mm)	SD	Mean power (W)	Variation (W)	SD	Mean LEED (J/cm)	Variation (J/cm)	SD
Saphenofemoral junction	6.70	(0.60 to 17.90)	2.72	-	-	-	-	-	-
Thigh	5.90	(0.30 to 23.60)	2.55	8.12	(4 to 10)	0.65	52.85	(13 to 160)	23.37
Knee	5.02	(0.30 to 23.10)	2.88	7.03	(4 to 10)	1.18	39.72	(6 to 102)	18.83
Leg	2.87	(0.30 to 6.80)	0.96	5.42	(2 to 8)	1.26	25.87	(4 to 83)	16.06
Total	-	-	-	-	-	-	45.90	(9 to 140)	20.60

Mean Ø = Mean Diameter; SD = Standard Deviation; LEED = Linear Intravenous Energy Density.

**Table 4:** Preoperative and transoperative data for the small saphenous vein.

	Small saphenous vein								
	Preoperative			Transoperative					
	Mean Ø (mm)	Variation (mm)	SD	Mean power (W)	Variation (W)	SD	Mean LEED (J/cm)	Variation (J/cm)	SD
Saphenopopliteal junction	5.31	(0.50 to 11.40)	2.69	-	-	-	-	-	-
Proximal leg	5.02	(0.50 to 19.30)	2.77	7.18	(5 to 9)	0.95	46.86	(15 to 111)	20.40
Distal leg	2.72	(0.30 to 5.10)	0.78	6.34	(4 to 8)	1.47	35.72	(5 to 111)	22.55
Total	-	-	-	-	-	-	44.07	(15 to 111)	19.46

Mean Ø = Mean Diameter; SD = Standard Deviation; LEED = Linear Intravenous Energy Density.

Patients’ complaints during the postoperative period were noted. Just 15.3% suffered pain, which was controlled using simple analgesic medication; 31.7% had paresthesia; 13.9% had edema; and 3.1% had ecchymosis at the 7-day follow-up consultation. There were 6 (2.1%) cases of DVT and all patients were treated in outpatients with Rivaroxaban.

Three of these patients merit description in greater detail. One had subacute DVT in the iliac-femoral-popliteal territory; the second developed DVT in the gastrocnemius vein with symptomology and prior history of bilateral popliteal cyst; and the third had DVT in a posterior tibial vein that was identified with control Doppler ultrasonography at 1 month and did not manifest any signs or symptoms. The other three cases involved gastrocnemius veins and had pain and edema in the calf. The three (1.0%) patients who developed EHIT were classified as type II, in which the thrombus extends beyond the SFJ, with cross-sectional diameter less than 50% of the lumen of the femoral vein. All were treated in outpatients with Rivaroxaban and ultrasound follow-up until resolution of the thrombus. Postoperative complications are listed in Table 5.

**Table 5:** Postoperative complications.

	n (%)
Paresthesia	91 (31.7%)
Pain	44 (15.3%)
Edema	40 (13.9%)
Ecchymosis	9 (3.1%)
DVT	6 (2.1%)

DVT = deep venous thrombosis; EHIT = endothermal heat-induced thrombosis.

During the follow-up period, all patients underwent control Doppler ultrasonography at 30 days, 6 months, and 12 months. At 30 days, 354 GSVs had been totally occluded (98.9% success) and 84 SSVs had undergone total occlusion (100% success). At 1 year, the success rates had fallen to 94.4% of GSVs and 96.4% of SSVs. Reflux in the GSV was observed during the three follow-up periods, reaching 14 (3.9%) cases at the end of 1 year. In contrast, there was only one (1.1%) case of SSV reflux at the 1-year control. The follow-up data are summarized in Table 6.

**Table 6:** Follow-ups with Doppler ultrasonography during the postoperative period.

	30 days		6 months		1 year	
	Reflux	Recanalization	Reflux	Recanalization	Reflux	Recanalization
GSV	4 (1.1%)	7 (1.9%)	7 (1.9%)	13 (3.6%)	14 (3.9%)	20 (5.6%)
SSV	0	0	0	0	1 (1.1%)	3 (3.6%)

GSV = Great Saphenous Vein; SSV = Small Saphenous Vein.

## DISCUSSION

Treatment of superficial venous insufficiency with EVLT has been in use for more than 15 years and proven to be an excellent option because of the high rates of safety, efficacy, and patient satisfaction when compared to other surgical techniques.^17^^-^19 If we compare it to conventional surgery, EVLT is a less invasive technique that can achieve better esthetic results while maintaining the effectiveness of conventional stripping.^1^^,^^12^ However, it is expensive and, because of this, is not accessible to all patients and is not available on the Brazilian National Health Service (SUS - Sistema Único de Saúde).^20^

The major questions related to use of lasers are the best type of fiber, the most appropriate wavelength, and the ideal LEED to be administered to each area. A variety of wavelengths are used to perform EVLT (810, 940, 980, 1,320, 1,470, and 1,940 nm).^21^ The different types of laser fiber administer energy to the vessel in different ways. Examples include conventional, tulip, nevertouch^TM^, and radial fibers.^22^

The 1,470 nm laser with radial fiber used in all of the patients in this study can emit energy directly to the vein wall in a radial pattern, enabling it to attain a larger area with a lower probability of intercurrent conditions than other fibers.^22^^,^^23^ This wavelength has a greater affinity for water than for hemoglobin. This leads to generation of a system of steam bubbles, heating the vein wall without the need for direct radiation, facilitating a higher success rate.^22^^,^^24^^,^^25^ Studies comparing the dispersal of 1,470 nm radial fiber laser with the 980 nm laser have shown that the first of these has certain advantages: it requires less energy to achieve adequate ablation and it is associated with fewer injuries to neighboring structures and, consequently, lower rates of postoperative complications. This enables patients to return to their routines more quickly, with venous reflux resolved.^24^^,^^26^

Identifying the ideal LEED is the key element in the success of this technique. A very high LEED has greater ablation power, but increases the likelihood of injuries to adjacent structures. In turn, if LEED is too low, it may result in insufficient energy being administered, increasing the likelihood of treatment failure and relapses. Several publications state that it is necessary to apply LEED from 65 to 100 J/cm to achieve adequate occlusion and fibrosis of the vein, with success rates from 90 to 100% at 1 year follow-up.^25^^-^28 However, a meta-analysis by Malskat et al.^17^ showed no significant difference in occlusion rates between groups with LEED > 50 J/cm and ≤ 50 J/cm. This discrepant information is explained by Proebstle et al.,^29^ who discuss the fluence (J/cm^2^) administered to the vessel lumen as a factor with potential impact on definition of the quantity of energy administered, taking into consideration the diameter of the vein treated along its entire length. Nevertheless, fluence is a very difficult variable to assess in all patients and there are few comparisons between studies. For this reason, the linear energy value (LEED) was used in the present study. The mean values were 45.90 J/cm for GSVs and 44.07 J/cm for SSVs, achieving success rates of 94.4% and 96.4%, respectively, at 12 months. It is possible that the reason for these high success rates lies in the use of Doppler ultrasonography in all of the patients assessed to conduct individualized analyses of the vein diameters of different segments and to control the result in real time.

The objective of treatment is total occlusion of incompetent superficial veins without injuring other structures, avoiding pain, pigmentation, paresthesia, and ecchymosis.^28^^,^^30^ These adverse effects are highly subjective and difficult to quantify. Even employing prospective protocols and data collection, retrospective analysis of these variables was very imprecise. In this study, there was a high prevalence of complaints of local “dormancy”, which was higher than rates reported in previous studies.^10^^,^^24^^,^^28^ This may have occurred for a number of reasons: use of ablation in distal segments, not employing tumescence, exeresis of varicose collaterals during the same session, administration of conventional sclerotherapy and/or sclerotherapy with dense foam, and even because there was a specific question on the postoperative control protocol. On the other hand, it was impossible to estimate the duration of this symptom, because the question was only posed once, at the 7-day follow-up. It is believed that numbness had resolved by later follow-ups, since this variable was not recorded again.^31^^,^^32^ This is considered a weakness of the study protocol.

A number of explanations have been proposed for the relationship between tumescence and lower rates of paresthesia.^10^ However, this variable could not be evaluated in the present study because the technique was used infrequently (23.3%). Erzinger et al.^10^ made this comparison and found that 7 days after surgery, paresthesia was significantly less frequent among patients in whom the tumescence technique had been used. However, at 30-day follow-up, the frequency of this complaint had reduced and was similar in the group that had not received tumescence, confirming the assertion above that these complaints had disappeared by later follow-ups.

Multiple perforations of the saphenous vein, injuries to the vein wall when advancing or tractioning the fiber, exeresis of varicose collaterals, and the tumescence technique are possible causes of the appearance of ecchymosis. This would explain the frequency of this sign among the patients in the present study. Venous thromboembolism (VTE) is one of the complications of invasive procedures, characterized by formation of acute thrombi in the deep vein system, primarily in lower limbs. The hospital’s VTE prophylaxis protocol evaluates risk factors such as age ≥ 40 years, scale of the surgery, prior VTE, known thrombophilias, limitations to mobility, and others to indicate use of anticoagulants. In this study, 2.1% of the patients developed DVT, which is consistent with other publications that report rates from 0.3 to 3.1%.^13^^,^^33^ The patient who developed iliac-femoral-popliteal DVT was later investigated to identify conditions that could have contributed to the condition and the anticoagulant lupus antibody was detected. If this finding had been predicted, the patient would have been classified as high risk, prophylaxis would have been prescribed, and the complication might have been avoided. Another case highlighted the importance of the Doppler ultrasonography examination at 1 month, since it enabled diagnosis and treatment of DVT in a low risk patient who was asymptomatic. None of the patients in this sample who developed DVT had been given prophylaxis. Another possible complication of EVLT is formation of thrombi close to the SFJ, known as EHIT.^33^^,^^34^ In view of the possibility of migration of these thrombi to the deep veins, with progression to DVT, ambulatory anticoagulant treatment with Rivaroxaban was administered in all 3 cases (1.0%) of type II EHIT. According to the literature, the prevalence of this complication is in the range of 0.9 to 6.4%^33^ and can be attributed to the need to maintain a distance of 2.5 cm or more from the SFJ to the initial point of ablation.^34^ This information was not recorded on the data collection protocol and so a more detailed analysis could not be conducted. Another explanation, discussed by Kane et al.,^35^ is linked to the higher prevalence of EHIT among patients who have a GSV with diameter > 7.5 mm, which was the case of one of the patients with EHIT (GSV = 9.8 mm).

One of the strengths of this study is provision of information on the preoperative diameters of the patients’ GSVs and SSVs, enabling calculation of the power of the laser and the LEED administered, segmenting by area and providing more specific details on the surgical technique employed. On the other hand, the study’s limitations result from failure to include information in the data collection instrument related to comorbidities, medications in use, and distance from initial point of ablation to the SFJ and SPJ. During the follow-up period, the study could also have benefited from active control of subjective complaints and administration of a questionnaire to assess patient satisfaction. These corrections have been incorporated into a new protocol to improve future studies.

## CONCLUSIONS

Use of the 1,470 nm diode laser proved to be safe, with low rates of complications, and achieved a high level of efficacy for resolution of venous reflux in incompetent superficial veins.
