# Supplementary material for: Use of 1,470 nm laser for treatment of superficial venous insufficiency
Source: J Vasc Bras. 2021 Jun 25;20:e20200244. doi: 10.1590/1677-5449.200244 (PMC8276655; doi:10.1590/1677-5449.200244)
Supplement: Translation

# Translation: Uso do *laser* de 1.470 nm para o tratamento de insuficiência venosa superficial

**Authors:** Manuella Bernardo Ferreira, Gilberto do Nascimento Galego, Nazaré Otília Nazário, Rafael Narciso Franklin, Pierre Galvagni Silveira, Cristiano Torres Bortoluzzi, Daniel Ishikawa, Fernando Wolf

**Affiliations:** 1 Universidade do Sul de Santa Catarina – UNISUL, Palhoça, SC, Brasil.; 2 Universidade Federal de Santa Catarina – UFSC, Florianópolis, SC, Brasil.; 3 Clínica Coris Medicina Vascular do Baía Sul Medical Center, Florianópolis, SC, Brasil.

**Keywords:** insuficiência venosa, varizes, angioplastia a *laser*, técnicas de ablação

## Abstract

**Contexto:**

Existem diversas formas de tratamento de varizes de membros inferiores. Entre elas, destaca-se o uso do *laser* diodo de 1.470 nm. Essa técnica proporciona aos pacientes uma cirurgia em regime ambulatorial, com retorno precoce à atividade ocupacional, bom resultado estético e baixo índice de complicações. No entanto, ainda se discute exaustivamente variáveis como comprimento de onda do *laser*, potência aplicada em cada área, tipo de fibra, necessidade ou não de tumescência e densidade de energia endovenosa linear.

**Objetivos:**

Analisar os resultados do tratamento da insuficiência venosa superficial com *laser* diodo de 1.470 nm.

**Métodos:**

Estudo retrospectivo, realizado em uma clínica privada de um hospital privado em Florianópolis a partir de dados colhidos prospectivamente. As amostras eram de 287 pacientes submetidos à cirurgia para tratamento da insuficiência venosa superficial com *laser* diodo de 1.470 nm, de janeiro de 2016 a dezembro de 2018, totalizando 358 veias safenas magnas e 84 veias safenas parvas tratadas.

**Resultados:**

A taxa de oclusão total após 12 meses de cirurgia foi de 94,4%, com densidade de energia endovenosa linear média de 45,90 J/cm nas veias safenas magnas e de 96,4% com densidade de energia endovenosa linear média de 44,07 J/cm nas veias safenas parvas.

**Conclusões:**

No período acompanhado, o *laser* diodo de 1.470 nm mostrou-se um tratamento seguro, muito efetivo e com baixas taxas de complicações (dor, edema, equimose, trombose venosa profunda e trombose induzida pelo calor endovenoso).

## INTRODUÇÃO

A insuficiência venosa crônica (IVC) é definida como o conjunto de manifestações clínicas em decorrência de alterações hemodinâmicas como refluxo e/ou obstrução do sistema venoso periférico (superficial e/ou profundo), geralmente dos membros inferiores^1^. As estimativas são de que metade da população mundial possui veias reticulares e telangiectasias em membros inferiores, e 25% apresentam veias varicosas maiores e mais visíveis^2^. No Brasil, a prevalência de varizes varia de 41,2 a 62,7% no sexo feminino e de 13,9 a 37,9% no masculino^3^. Segundo o Ministério da Saúde, a repercussão socioeconômica causada pela IVC ocupa a 14ª posição entre as 50 doenças que causam afastamento do trabalho^4^^,^^5^.

Essa alta prevalência está associada a envelhecimento, obesidade, história familiar, sexo feminino (5:1), mulheres caucasianas, uso de anticoncepcional oral, terapia de reposição hormonal e atividades laborais em ortostatismo^2^^,^^3^. O impacto na redução da qualidade de vida aliado a cronicidade e subjetividade de queixas e sintomas como dor, sensação de peso, inchaço, câimbras, entre outros necessita de avaliação clínica para estabelecer a sua distribuição anatômica e quantificar os efeitos hemodinâmicos para definir a gravidade da doença^1^^,^^6^^,^^7^.

A manifestação mais comum da IVC são as veias varicosas, que se iniciam em pontos de refluxo, como na junção safeno-femoral (JSF), na junção safeno-poplítea (JSP) ou nos sistemas de veias perfurantes devido a insuficiência valvular e/ou dilatação das paredes venosas. Esses eventos ocasionam estase, deficiência na drenagem e hipertensão venosa culminando em edema, déficit de nutrição tecidual e alterações cutâneas, que consequentemente favorecem a instalação de processos inflamatórios e infecciosos, aumentando o risco de tromboses venosas e lesões como lipodermatoesclerose e úlceras venosas^2^^,^^7^^-^9.

Para evitar a progressão da doença, a ligadura e extração da veia safena magna (VSM) e/ou parva (VSP) combinadas com a exérese das varizes e ligadura das veias perfurantes insuficientes tem sido o padrão de intervenção^10^. Com o avanço terapêutico, surgiram novas abordagens com técnicas menos invasivas e resultados equivalentes, como o tratamento com *laser* endovenoso (EVLT [*endovenous laser treatment*]), radiofrequência, escleroterapia com espuma combinadas ou não com flebectomia e escleroterapia líquida^10^^-^12.

Entre elas, o EVLT possibilita o tratamento de pacientes em regime ambulatorial, com retorno precoce à atividade ocupacional, melhor resultado estético e baixo índice de complicações. Essa técnica utiliza o *laser* endovenoso para gerar ablação venosa e tratar o refluxo nesse território^10^^,^^13^^-^16. No entanto, discute-se incessantemente os efeitos dos tipos de fibras, comprimentos de onda e potência do *laser*, bem como a densidade de energia endovenosa linear (LEED [*linear endovenous energy density*]) aplicada para obter o melhor resultado terapêutico e minimizar as complicações operatórias, como: dor, equimose, tromboflebite, trombose induzida pelo calor endovenoso (EHIT [*endothermal heat-induced thrombosis*]) e trombose venosa profunda (TVP). Frente ao contexto descrito, este estudo visa descrever os resultados do tratamento da insuficiência venosa superficial com o uso do *laser* diodo de 1.470 nanômetros (nm).

## METODOLOGIA

Estudo transversal descritivo realizado a partir da base de dados de uma clínica privada, de referência e especializada em tratamentos endovasculares, localizada em um hospital privado em Florianópolis, no estado de Santa Catarina. A amostra foi constituída por 287 pacientes submetidos à cirurgia de varizes de membros inferiores com *laser* diodo de 1.470 nm de fibra radial, no período de janeiro de 2016 a dezembro de 2018. A amostra de 280 pacientes foi considerada suficiente para medir uma prevalência esperada de 18% de maior severidade na classificação clínica, etiológica, anatômica e patológica (CEAP [*Clinical-Etiology-Anatomy-Physiopathology*]), com erro tolerado de +/- 5%, intervalo de confiança de 95% e poder de 80%. Foram incluídos os pacientes maiores de 18 anos, de ambos os sexos, com diagnóstico e indicação de tratamento cirúrgico para varizes de membros inferiores unilateral ou bilateral, pertencentes à classe C2 a C6 da CEAP, com no mínimo dois exames de eco-Doppler de controle no pós-operatório e realização de ablação na VSM e/ou VSP com o *laser* de 1.470 nm. Foram excluídos os pacientes com diagnóstico prévio de TVP.

O protocolo utilizado consistiu em três etapas. Na primeira, pré-operatória, realizou-se a avaliação clínica e com eco-Doppler, permitindo a classificação pela CEAP, a localização do refluxo venoso e o diâmetro dos vasos acometidos. Na segunda etapa, transoperatória, confirmaram-se as informações coletadas no pré-operatório, e, com o uso do eco-Doppler, foi determinado o local da punção venosa. Acompanhou-se o trajeto da fibra e o resultado da ablação. Posteriormente, a potência do *laser* de 1.470 nm foi definida pelo cirurgião vascular, com base no diâmetro do vaso insuficiente e na proximidade com a pele. A fibra foi tracionada com velocidade constante, levando à liberação de energia e ablação venosa, ao mesmo tempo em que se realizou a compressão manual ou com o transdutor do eco-Doppler para aproximar as paredes da veia à fibra. Em alguns casos, optou-se pela realização da técnica de tumescência, que consiste na infiltração de soro fisiológico 0,9% resfriado com auxílio do eco-Doppler em toda a extensão da veia safena até que fique colabada, com aspecto esbranquiçado ou sinal da pérola. No mesmo ato operatório, foi realizada a ressecção de colaterais e/ou escleroterapia. Ao fim da ablação, a energia total dissipada foi registrada no protocolo, assim como a extensão do vaso tratado, visando ao cálculo da LEED (Joules/cm). A dissecção e ligadura da crossa da safena ficaram a critério do cirurgião, dependendo do diâmetro da veia safena em nível da JSF e JSP e de dados na visualização pelo eco-Doppler. Antes do término do procedimento, utilizou-se o eco-Doppler para verificar a perviedade de veia femoral e poplítea e confirmar a ablação da safena. Todos os pacientes realizaram o procedimento em regime de centro cirúrgico, sob raquianestesia.

No terceiro estágio, de acompanhamento, orientou-se a deambulação precoce e a utilização de meia elástica 7/8 e/ou enfaixamento completo com atadura do membro inferior. Entre o sétimo e o décimo dia de pós-operatório, foi feita a pesquisa de equimose, dor, parestesia e edema e indicou-se a troca pelas meias elásticas de compressão média do tipo 3/4. Os seguimentos com eco-Doppler foram realizados com 30 dias, 6 meses e 12 meses do pós-operatório para avaliação de *status* hemodinâmico das safenas e identificação de complicações (TVP, EHIT etc.). Na suspeita de TVP ou a critério clínico (dor e edema desproporcional, edema de aparecimento súbito, evolução desfavorável e outros), o exame de eco-Doppler foi antecipado. Considerou-se sucesso no tratamento quando houve oclusão total do segmento tratado. Durante o acompanhamento, a evidência de fluxo num segmento venoso previamente definido como ocluído no eco-Doppler anterior representou a falha no tratamento e foi descrito como recanalização.

Os dados foram compilados no *software* Excel^®^ 12.63 (Microsoft Corporation, Washington, EUA) e importados para o Statistical Package for the Social Sciences 18.0 (IBM, Nova Iorque, EUA) para análise estatística. Na análise descritiva, as variáveis qualitativas foram apresentadas como frequência simples e relativa, e as variáveis quantitativas foram apresentadas através de medidas de tendência central e suas respectivas medidas de dispersão. O projeto foi apreciado e aprovado pelo Comitê de Ética em Pesquisa da Universidade do Sul de Santa Catarina, sob parecer consubstanciado de nº 3.494.758.

## RESULTADOS

Entre janeiro de 2016 e dezembro de 2018, 287 pacientes foram submetidos ao tratamento de varizes com o *laser* diodo de 1.470 nm de fibra radial. A média de idade da população em estudo foi de 52,36 anos, sendo 219 (76,3%) do sexo feminino. Conforme indicação, a necessidade de abordagem venosa foi individualizada para cada paciente. Entre as intervenções mais prevalentes, se destacam a ablação de apenas uma VSM em 136 (47,4%) casos e a ablação das duas VSMs em 78 (27,2%). Todas as características demográficas constam na Tabela 1.

**Tabela 1:** Dados demográficos dos pacientes (n = 287).

Média de idade (anos)	52,36 (19 a 77)
Gênero	
Feminino	219 (76,3%)
Masculino	68 (23,7%)
ASA	
1	101 (38,4%)
2	161 (61,2%)
3	1 (0,4%)
Tipo de cirurgia	
1 x VSM	136 (47,4%)
2 x VSM	78 (27,2%)
1 x VSP	17 (5,9%)
2 x VSP	7 (2,4%)
1 x VSM e 1 x VSP	29 (10,1%)
1 x VSM e 2 x VSP	2 (0,7%)
2 x VSM e 2 x VSP	2 (0,7%)

ASA = *American Society of Anesthesiologists*; VSM = Veia Safena Magna; VSP = Veia Safena Parva.

Quanto à CEAP, observou-se que na maioria das vezes variavam de C2 a C4, sendo a categorização C3, que possui edema associado, a mais frequente. Essas informações estão elucidadas na Tabela 2. Os dados de 30 pacientes foram omitidos visto que a classificação não constava no prontuário.

**Tabela 2:** Classificação clínica pré-operatória (CEAP).

	C1	C2	C3	C4	C5	C6
Geral (n = 442)	0	77	274	52	4	5
VSM (n = 358)	0	61	224	41	3	4
VSP (n = 84)	0	16	50	11	1	1

VSM = Veia Safena Magna; VSP = Veia Safena Parva.

Os dados encontrados no exame de eco-Doppler pré-operatório são mostrados nas Tabelas 3 e 4 e estão descritos por região. Nelas, também estão detalhadas a potência média e a LEED necessária por área para fazer a ablação durante a cirurgia. A VSM, na altura da coxa, apresentava diâmetro médio de 5,90 mm, e foi aplicada potência média de 8,12 W e calculou-se LEED média de 52,85 J/cm. Ao contabilizar a energia e extensão totais usadas na ablação da VSM, a LEED média foi de 45,90 J/cm. Já em relação a VSP, o diâmetro médio na perna proximal foi de 5,02 mm, usou-se a potência média de 7,18 W e a LEED média foi de 46,86 J/cm. A LEED média total na VSP foi de 44,07 J/cm.

**Tabela 3:** Dados pré-operatórios e transoperatórios da veia safena magna.

	Veia safena magna								
	Pré-operatório			Transoperatório					
	Ø médio (mm)	Variação (mm)	DP	Potência média (W)	Variação (W)	DP	LEED média (J/cm)	Variação (J/cm)	DP
Junção safeno-femoral	6,70	(0,60 a 17,90)	2,72	-	-	-	-	-	-
Coxa	5,90	(0,30 a 23,60)	2,55	8,12	(4 a 10)	0,65	52,85	(13 a 160)	23,37
Joelho	5,02	(0,30 a 23,10)	2,88	7,03	(4 a 10)	1,18	39,72	(6 a 102)	18,83
Perna	2,87	(0,30 a 6,80)	0,96	5,42	(2 a 8)	1,26	25,87	(4 a 83)	16,06
Total	-	-	-	-	-	-	45,90	(9 a 140)	20,60

Ø médio = Diâmetro Médio; DP = Desvio Padrão; LEED = Densidade de Energia Endovenosa Linear.

**Tabela 4:** Dados pré-operatórios e transoperatórios da veia safena parva.

	Veia safena parva								
	Pré-operatório			Transoperatório					
	Ø médio (mm)	Variação (mm)	DP	Potência média (W)	Variação (W)	DP	LEED média (J/cm)	Variação (J/cm)	DP
Junção safeno-poplítea	5,31	(0,50 a 11,40)	2,69	-	-	-	-	-	-
Perna proximal	5,02	(0,50 a 19,30)	2,77	7,18	(5 a 9)	0,95	46,86	(15 a 111)	20,40
Perna distal	2,72	(0,30 a 5,10)	0,78	6,34	(4 a 8)	1,47	35,72	(5 a 111)	22,55
Total	-	-	-	-	-	-	44,07	(15 a 111)	19,46

Ø médio = Diâmetro Médio; DP = Desvio Padrão; LEED = Densidade de Energia Endovenosa Linear.

No pós-operatório, fez-se o registro das queixas dos pacientes. Apenas 15,3% apresentavam dor, que foi controlada com uso de medicação analgésica simples; 31,7% cursaram com parestesia; 13,9%, com edema; e 3,1%, com equimose na avaliação de 7 dias. Foram evidenciados 6 (2,1%) casos de TVP, e todos os pacientes receberam tratamento ambulatorial com Rivaroxabana.

Desses pacientes, três merecem maiores detalhes. Um apresentou TVP subaguda no território ilíaco-femoro-poplíteo; o segundo caso desenvolveu TVP em veia gastrocnêmia com sintomatologia e história pregressa de cisto poplíteo bilateral; e o terceiro cursou com TVP em veia tibial posterior e não apresentou nenhum sinal ou sintoma, sendo identificada no eco-Doppler de controle de 1 mês. Os outros três casos envolviam veias gastrocnêmias e apresentaram dor e edema em panturrilha. Os três (1,0%) pacientes que cursaram com EHIT foram classificados como tipo II, em que a extensão do trombo se prolonga para além da JSF, com diâmetro de secção transversal menor que 50% da luz da veia femoral. Todos receberam tratamento ambulatorial com Rivaroxabana e acompanhamento ecográfico até a resolução do trombo. As complicações pós-operatórias estão discriminadas na Tabela 5.

**Tabela 5:** Complicações pós-operatórias.

	n (%)
Parestesia	91 (31,7%)
Dor	44 (15,3%)
Edema	40 (13,9%)
Equimose	9 (3,1%)
TVP	6 (2,1%)
EHIT	3 (1,0%)

TVP = trombose venosa profunda; EHIT = trombose induzida pelo calor endovenoso.

Durante o período de seguimento, todos os pacientes foram submetidos a um eco-Doppler de controle em 30 dias, 6 meses e 12 meses. Aos 30 dias, 354 VSMs apresentavam oclusão total (98,9% de sucesso) e 84 VSPs, oclusão total (100% de sucesso). Já em 1 ano, a taxa de sucesso foi para 94,4% nas VSMs e 96,4% nas VSPs. O aparecimento de refluxo na VSM foi evidenciado nos três períodos de seguimento, totalizando 14 (3,9%) casos ao final de 1 ano. No entanto, na VSP, evidenciou-se somente um (1,1%) caso de refluxo no controle de 1 ano. Os dados de seguimento estão sintetizados na Tabela 6.

**Tabela 6:** Seguimento com eco-Doppler no pós-operatório.

	30 dias		6 meses		1 ano	
	Refluxo	Recanalização	Refluxo	Recanalização	Refluxo	Recanalização	
VSM	4 (1,1%)	7 (1,9%)	7 (1,9%)	13 (3,6%)	14 (3,9%)	20 (5,6%)
VSP	0	0	0	0	1 (1,1%)	3 (3,6%)

VSM = Veia Safena Magna; VSP = Veia Safena Parva.

## DISCUSSÃO

O tratamento da insuficiência venosa superficial com o EVLT tem sido utilizado há mais de 15 anos e mostra-se uma excelente alternativa devido à alta taxa de segurança, efetividade e satisfação dos pacientes quando comparada a outras técnicas cirúrgicas^17^^-^19. Ao confrontarmos com a cirurgia convencional, o EVLT é uma técnica menos invasiva, que consegue melhor resultado estético e mantém a efetividade da abordagem convencional^1^^,^^12^. Porém, apresenta custo mais elevado e, por isso, não é uma técnica acessível a todos os pacientes nem está disponível no Sistema Único de Saúde^20^.

As grandes questões relacionadas com o uso do *laser* são definição do melhor tipo de fibra, qual o comprimento de onda mais adequado a ser utilizado e conhecimento da LEED ideal a ser aplicada em cada área. Existe uma variedade de comprimentos de onda que são utilizados para a realização do EVLT (810, 940, 980, 1.320, 1.470 e 1.940 nm)^21^. Ao mesmo tempo, os diferentes tipos de fibra do *laser* possibilitam que a energia seja aplicada no vaso de maneiras distintas. Como exemplos, há a convencional, a tulipa, a *nevertouch* ^TM^ e a radial^22^.

O *laser* de fibra radial de 1.470 nm usado em todos os pacientes deste estudo tem a capacidade de emitir a energia diretamente na parede da veia em dispersão radial, conseguindo atingir uma área maior com menor probabilidade de intercorrências do que outras fibras^22^^,^^23^. Esse comprimento de onda possui maior afinidade pela água do que pela hemoglobina. Isso acarreta a geração de um sistema de bolhas de vapor, promovendo o aquecimento da parede venosa sem a necessidade de radiação direta, favorecendo maior taxa de sucesso^22^^,^^24^^,^^25^. Os estudos que comparam a dispersão do *laser* com fibra radial de 1.470 nm com o de 980 nm mostram algumas vantagens do primeiro: necessidade de menos energia para obtenção da ablação adequada, redução de aparecimento de lesão de estruturas vizinhas e, consequentemente, menor taxa de complicações pós-operatórias. Isso se traduz em retorno mais rápido do paciente à rotina normal, com a resolução do refluxo venoso^24^^,^^26^.

Encontrar a LEED ideal é o ponto-chave para o sucesso da técnica. A LEED muito elevada tem maior poder de ablação, porém, aumenta a possibilidade de lesões de estruturas adjacentes. Já a LEED baixa pode não entregar uma energia suficiente, aumentando a chance de insucesso e recidivas. Diversas literaturas trazem a necessidade da aplicação de uma LEED entre 65 e 100 J/cm para se obter oclusão e fibrose adequada da veia, com taxas de sucesso entre 90 e 100% em um ano de acompanhamento^25^^-^28. Entretanto, a metanálise de Malskat et al.^17^ mostrou não haver diferença significativa na taxa de oclusão nos grupos com LEED > 50 J/cm e ≤ 50 J/cm. Essa discrepância de informações é justificada por Proebstle et al.^29^, que trazem a discussão da fluência (J/cm^2^) aplicada à luz do vaso como um fator com potencial de impacto na definição da quantidade de energia aplicada, levando em consideração o diâmetro da veia tratada em todo seu trajeto. De fato, a fluência é uma variável difícil de ser avaliada em todos os pacientes e pouco comparada nos estudos. Por essa razão, utilizou-se o valor da energia linear no presente estudo, a LEED. Ela teve valor médio de 45,90 J/cm para a VSMs e de 44,07 J/cm para as VSPs, mantendo a taxa de êxito, em 12 meses, de 94,4% e 96,4%, respectivamente. Talvez a explicação para essa expressiva taxa de sucesso esteja no uso do eco-Doppler para uma análise individualizada do diâmetro da veia nos vários segmentos e controle do resultado em tempo real de todos os pacientes avaliados.

O objetivo do tratamento é a oclusão total das veias superficiais insuficientes sem provocar lesão em outras estruturas evitando dor, pigmentação, parestesia e equimose^28^^,^^30^. Esses efeitos adversos são bastante subjetivos e de difícil quantificação. Apesar da utilização de protocolo e coleta de dados de forma prospectiva, a análise retrospectiva dessas variáveis foi bastante imprecisa. Neste estudo, identificou-se uma alta prevalência da queixa de “dormência” local, que foi maior do que a registrada em estudos anteriores^10^^,^^24^^,^^28^. Isso pode ter ocorrido por várias razões: realização de ablação em segmentos distais, não aplicação da tumescência, exérese de colaterais varicosas no mesmo tempo operatório, realização de escleroterapia convencional e/ou com espuma densa e até mesmo pela pergunta ativa existente no protocolo de controle pós-operatório. Por outro lado, não foi possível estimar a duração desse sintoma, pois questionou-se somente uma vez, na avaliação de 7 dias. Entende-se que a sua resolução tenha ocorrido nos controles subsequentes, uma vez que esse dado não foi mais registrado^31^^,^^32^. Isso foi apontado como falha no protocolo do estudo.

São colocadas algumas explicações que relacionam a realização de tumescência com a diminuição da ocorrência de parestesia^10^. Entretanto, essa variável não pode ser avaliada neste estudo pelo baixo uso (23,3%) da técnica. Erzinger et al.^10^ fizeram essa comparação, e, no controle de 7 dias após a cirurgia, a parestesia foi significativamente menor nos pacientes nos quais se aplicou a técnica de tumescência. Porém, no seguimento de 30 dias, a frequência da queixa diminuiu e ficou semelhante às queixas do grupo em que não foi realizada a tumescência, corroborando com a discussão anterior de que essas queixas desapareceram nos controles mais tardios.

Múltiplas perfurações da veia safena, lesão da parede venosa durante a progressão da fibra, exérese de colaterais varicosas e a própria tumescência podem explicar o aparecimento de manchas equimóticas. Isso explica a constatação frequente desse sinal nos pacientes do presente estudo. O tromboembolismo venoso (TEV) é uma das complicações de procedimentos invasivos, caracterizado pela formação de trombos agudos no sistema venoso profundo, principalmente em membros inferiores. O protocolo de profilaxia de TEV do hospital avalia fatores de risco, como idade ≥ 40 anos, porte da cirurgia, TEV prévio, trombofilia conhecida, limitação de mobilidade, entre outros para indicar o uso de anticoagulantes. Neste estudo, 2,1% dos pacientes evoluíram com TVP, corroborando com outras literaturas que trazem valores de 0,3 a 3,1%^13^^,^^33^. A paciente que cursou com TVP ilíaco-femoro-poplítea foi posteriormente investigada para condições que poderiam ter contribuído para a instalação deste quadro, e, nela, foi encontrado o anticorpo anticoagulante lúpico. Se tal achado tivesse sido previsto e a paciente classificada como alto risco, haveria indicação de profilaxia e a complicação poderia ter sido evitada. Em outro caso, ressaltou-se a importância da avaliação com o eco-Doppler em 1 mês, o que possibilitou o diagnóstico de TVP e tratamento de uma paciente de baixo risco e assintomática. Todos os pacientes que desenvolveram TVP, nesta amostra, não realizaram profilaxia. Outra complicação do EVLT é a formação de trombos próximos a JSF, denominada EHIT^33^^,^^34^. Devido a possibilidade de migração desses trombos para as veias profundas com evolução para uma TVP, foi realizado o tratamento anticoagulante ambulatorial com Rivaroxabana nos 3 casos (1,0%) de EHIT tipo II. Segundo a literatura, a prevalência dessa complicação está na faixa de 0,9 a 6,4%^33^ e pode ser atribuída à necessidade de manter uma distância de 2,5 cm ou mais da JSF do início da ablação^34^. Essa informação não foi registrada no protocolo de coleta de dados e não possibilitou uma análise mais apurada. Outra justificativa, discutida por Kane et al.^35^, seria de uma maior prevalência de EHIT em pacientes que apresentam VSM com diâmetro > 7,5 mm, o que foi evidenciado em um dos pacientes com EHIT (VSM = 9,8 mm).

O diferencial deste estudo foi trazer a informação do diâmetro pré-operatório das VSMs e VSPs dos pacientes operados, possibilitando a determinação da potência do *laser* e a LEED utilizados, segmentando-os por área e caracterizando a técnica cirúrgica com maior especificidade. Por outro lado, as limitações do estudo consistem na falta de inclusão de informações no instrumento de coleta sobre comorbidades, medicamentos em uso e distância do início da ablação da JSF e da JSP. No período de seguimento, o estudo talvez se beneficiaria de um controle ativo das queixas subjetivas e da realização de um questionário para avaliação da satisfação do paciente. Essas correções foram incorporadas ao novo protocolo para otimização de estudos futuros.

## CONCLUSÃO

O uso do *laser* diodo de 1.470 nm mostrou-se seguro e com baixas taxas de complicações, mantendo alto índice de efetividade na resolução do refluxo venoso nas veias superficiais insuficientes.
